# Nitric oxide and cytokine production by glial cells exposed in vitro to neuropathogenic schistosome *Trichobilharzia regenti*

**DOI:** 10.1186/s13071-016-1869-7

**Published:** 2016-11-14

**Authors:** Tomáš Macháček, Lucie Panská, Hana Dvořáková, Petr Horák

**Affiliations:** Department of Parasitology, Faculty of Science, Charles University, Viničná 7, Prague 2, 12844 Czech Republic

**Keywords:** Astrocytes, Microglia, *Trichobilharzia regenti*, Avian schistosome, Neuroinfection, Nitric oxide, Proinflammatory cytokines, Anti-inflammatory cytokines, Cathepsin B

## Abstract

**Background:**

Helminth neuroinfections represent a serious health problem, but host immune mechanisms in the nervous tissue often remain undiscovered. This study aims at in vitro characterization of the response of murine astrocytes and microglia exposed to *Trichobilharzia regenti* which is a neuropathogenic schistosome migrating through the central nervous system of vertebrate hosts. *Trichobilharzia regenti* infects birds and mammals in which it may cause severe neuromotor impairment. This study was focused on astrocytes and microglia as these are immunocompetent cells of the nervous tissue and their activation was recently observed in *T. regenti*-infected mice.

**Results:**

Primary astrocytes and microglia were exposed to several stimulants of *T. regenti* origin. Living schistosomulum-like stages caused increased secretion of IL-6 in astrocyte cultures, but no changes in nitric oxide (NO) production were noticed. Nevertheless, elevated parasite mortality was observed in these cultures. Soluble fraction of the homogenate from schistosomulum-like stages stimulated NO production by both astrocytes and microglia, and IL-6 and TNF-α secretion in astrocyte cultures. Similarly, recombinant cathepsins B1.1 and B2 triggered IL-6 and TNF-α release in astrocyte and microglia cultures, and NO production in astrocyte cultures. Stimulants had no effect on production of anti-inflammatory cytokines IL-10 or TGF-β1.

**Conclusions:**

Both astrocytes and microglia are capable of production of NO and proinflammatory cytokines IL-6 and TNF-α following in vitro exposure to various stimulants of *T. regenti* origin. Astrocytes might be involved in triggering the tissue inflammation in the early phase of *T. regenti* infection and are proposed to participate in destruction of migrating schistosomula. However, NO is not the major factor responsible for parasite damage. Both astrocytes and microglia can be responsible for the nervous tissue pathology and maintaining the ongoing inflammation since they are a source of NO and proinflammatory cytokines which are released after exposure to parasite antigens.

**Electronic supplementary material:**

The online version of this article (doi:10.1186/s13071-016-1869-7) contains supplementary material, which is available to authorized users.

## Background

Invasion of the central nervous system (CNS) of mammals, including humans, by parasitic helminths is a well-recognized phenomenon. Apart from recruitment of peripheral leukocytes, astrocytes and microglia (i.e. CNS-resident glial cells) can be activated during the infection and exhibit antiparasitic effects [1, 2]. In this study, we examined the response of astrocytes and microglia to the neuropathogenic bird schistosome *Trichobilharzia regenti*.

*Trichobilharzia regenti* is widely distributed in Europe, e.g. Czech Republic [3], Denmark [4], France [5], Iceland [6] or Russia [7], and was also detected in Iran [8]. It uses anatid birds, e.g. ducks, as definitive hosts. They become infected by cercariae, freely swimming larvae emerging from lymnaeid snails which serve as intermediate hosts [3]. Apart from birds, *T. regenti* cercariae are able to penetrate the skin of accidental mammalian hosts, e.g. mice or humans. This may result in a skin allergic reaction known as cercarial dermatitis which is regarded as a re-emerging disease [9–11]. To penetrate the host’s skin, cercariae are equipped with proteases present in their excretory/secretory products (ESP; [12]), such as cysteine protease cathepsin B2 from post-acetabular glands that was shown to cleave skin proteins like collagen, keratin and elastin [13].

Contrary to human schistosomes, the newly transformed schistosomula of *T. regenti* avoid penetration into skin blood capillaries and rather enter peripheral nerves in host‘s limbs where they appear 1–1.5 day post-infection (dpi). Parasite migration in definitive hosts continues towards and via the spinal cord and the brain, and adult worms occur in nasal mucosa of ducks 13–14 dpi and lay eggs there [14, 15]. The invasion of the nervous system by *T. regenti* schistosomula is often accompanied by serious neurological malfunctions in birds that suffer from leg paralysis and balance disorders [16].

A different course of the infection is observed in mice. Although schistosomula are found in the lumbar spinal cord as early as two dpi and *medulla oblongata* may be invaded the day after in some individuals, most parasites stay localized in the thoracic and cervical spinal cord and the migration to the brain is exceptional [14, 16]. As recently demonstrated, schistosomula feed on the nervous tissue when they pass through the spinal cord [17]. A cysteine protease, cathepsin B1, the intestinal enzyme of schistosomula, may be responsible for digestion since it was shown to degrade myelin basic protein [18].

However, the development of *T. regenti* is suppressed in mice and schistosomula do not reach maturity. It was hypothesized that this is possibly due to the host immune response and/or the absence of some essential nutritional or stimulatory factors [19]. The supposed role of the host’s immunity in regulation of parasite migration is supported by experiments with immunocompetent and immunodeficient mouse strains. Immunodeficient mice display higher schistosomulum burden, the parasites also migrate faster in their CNS and reach brain hemispheres more often [14, 20]. Furthermore, the damaged schistosomula can be detected in the CNS from seven dpi in immunocompetent mice whereas in immunodeficient ones the parasite destruction appears two weeks later [17].

Research on the host immune response revealed a strong inflammatory cellular infiltration consisting of mononuclear cells, granulocytes, plasma cells and histiocytes, observed especially around the damaged schistosomula [20, 21]. Mononuclear cells present in the lesions were characterized as macrophages and CD3+ lymphocytes which likely cooperate in schistosomula destruction [17]. Consequently, CD3-deficient mice developed no or only mild inflammation which was accompanied by neurological symptoms as described above for bird hosts [17, 20].

Additionally, activation of astrocytes and microglia has recently been detected in the CNS of mice infected by *T. regenti* [17]. Activated microglia were observed in the migratory tracks of schistosomula and in the inflammatory lesions containing parasite residues. In addition, hypertrophy and activation of astrocytes located in the migratory tracks and in the proximity of schistosomula were seen, suggesting their role in immune response and/or reparation of the tissue [17]. Nevertheless, no functional assays evaluating the role of astrocytes and microglia in the host immune response have been performed yet.

In this study, we set up primary cultures of murine astrocytes and microglia and exposed them to various stimulants of *T. regenti* origin: living schistosomulum-like stages, soluble fraction of their homogenate and recombinant cathepsins B1.1 and B2. Forty-eight hours after the stimulation, we measured production of nitric oxide (NO) and proinflammatory cytokines, particularly interleukin (IL)-1β, IL-6, and tumor necrosis factor (TNF)-α, by the cells in order to assess their potential role in host immune response. To evaluate possible regulatory activities of astrocytes and microglia, we also analyzed their secretion of anti-inflammatory cytokines IL-10 and transforming growth factor (TGF)-β1.

## Methods

### Animal handling

Mice of C57BL/6J strain (Harlan Laboratories, Italy) were bred in the Centre for Experimental Biomodels (Charles University, First Faculty of Medicine) and their pups were used for preparation of glial cell cultures (see below). Parental animals were regularly examined according to FELASA recommendations.

### Glial cell cultures

Mice pups of C57BL/6J strain not older than 48 h were used for preparation of mixed glial cell cultures from which astrocytes and microglia were isolated according to established protocols [22, 23]. Briefly, brain was dissected out of cranium and meninges/vessels were removed carefully. After that, the tissue was mechanically disintegrated by pipetting up and down, and the resulting cell suspension was placed into a tissue culture flask coated with poly-L-lysine (5 μl/ml; Sigma-Aldrich, St. Louis, Missouri, USA). The cells were grown in RPMI 1640 (Lonza, Basel, Switzerland) supplemented by 10% fetal bovine serum (Thermo Fisher Scientific, Waltham, Massachusetts, USA), 2 mM glutamine (Lonza), 100 U/ml penicillin (Lonza) and 100 μg/ml streptomycin (Lonza) at 37 °C and 5% CO_2_. The medium was changed the next day after the culture establishment and then every 2–3 days. Approximately 1–2 days after the cells reached confluence, the flasks were placed in an orbital shaker and shaken for 6–8 h at 200 rpm at 37 °C.

To obtain microglia, supernatants from flasks after shaking were gathered in sterile 50 ml centrifugation tubes and centrifuged at 300× *g* and 4 °C; the centrifugation time was 1 min per milliliter of the suspension volume. The pellet was then resuspended in the medium and the cells were counted by Countess™ Automated Cell Counter (Invitrogen, Carlsbad, California, USA). Cells were seeded in cell culture treated 12-well plates (Nunc) at the density of 2.0 × 10^5^ cells/well. The plates were incubated (37 °C, 5% CO_2_) for 10 min to let microglia adhere. The wells were then washed three times with the medium and incubated for 48 h to settle down before stimulation.

The cells remaining in the cultivation flasks after shaking and harvesting microglia were trypsinized and centrifuged at 300× *g* and 4 °C; the centrifugation time was 1 min per milliliter of the suspension volume. The pellet was resuspended in medium and the cells were counted as described above and seeded in 12-well plates (Nunc) at the density of 2.0 × 10^5^ cells per well. Prior to stimulation, the cells represented mostly by astrocytes were let calm down for 48 h in the incubator.

### Cell culture purity

To verify identity of the cells in cultures, immunocytochemical staining was performed. The cells were fixed by freshly prepared 4% paraformaldehyde for 20 min, treated with 0.1% Triton X-100 for 5 min to permeabilize cell membranes and blocked by 10% normal goat serum (NGS) for 30 min. Afterwards, the cells were overnight incubated with rabbit polyclonal anti glial fibrillary acidic protein (GFAP, astrocyte marker) antibody (1:2000 in 10% NGS; Dako, Glostrup, Denmark) or rabbit polyclonal anti ionized calcium-binding adapter molecule 1 (Iba1, microglia marker) antibody (1:1000 in 10% NGS; Wako, Neuss, Germany) to detect astrocytes and microglia, respectively. Non-immunized rabbit immunoglobulin fraction (Dako) was used instead of the primary antibody as a negative control. Finally, the cells were incubated with goat anti rabbit Alexa Fluor 488 or 568 (1:2000 in PBS; Thermo Fisher Scientific) for 60 min, mounted in VectaShield medium with DAPI (Vector Labs, Burlingame, California, USA) and examined under a fluorescence microscope (Olympus BX51).

The purity of cultures was calculated as proportions of GFAP+ or Iba1+ cells in astrocyte or microglia cultures, respectively. A cross-examination detecting GFAP+ cells in microglia cultures and Iba1+ cells in astrocyte cultures was done in the same way. Five independent samples with at least 100 cells were counted for each analysis.

### Cell stimulation

Stimulation of astrocytes and microglia was conducted under sterile conditions in a laminar flow hood (ESCO). The medium itself (negative control) or supplemented by different stimulants (see Table 1 and the following section) was added to the cells which were then incubated at 37 °C and 5% CO_2_ for 48 h. After that, the supernatant was collected and concentration of NO was measured immediately whereas samples dedicated for cytokine analysis were stored at -80 °C until further processed.

**Table 1:** Stimulants tested in glial cell cultures

Stimulant	Concentration (per 1 ml/well)
Living schistosomulum-like stages of *T. regenti* (LS)	15 individuals
Soluble fraction of LS homogenate (HSF)	50 μg
Recombinant *T. regenti* cathepsin B1.1 (rTrCB1.1)	1 μg
Recombinant *T. regenti* cathepsin B2 (rTrCB2)	1 μg
Lipopolysaccharide from *Escherichia coli* 0127:B8 (LPS, Sigma-Aldrich), a positive control	0.5 μg

Five independent experiments were conducted with each stimulant. In all experiments, the stimulation of astrocytes was performed in triplicates while that of microglia in duplicates since 7.7-fold less microglia were yielded from mixed cultures (Additional file 1: Figure S1).

### Preparation of stimulants

Living schistosomulum-like stages of *T. regenti* (LS) were prepared by mechanical transformation of cercariae [24]. Briefly, the cercariae were collected after their release from the laboratory-reared intermediate snail host *Radix lagotis*; the release was provoked by illumination of the snails for 30–40 min. The cercariae were passed through a 23G-syringe needle 20 times to detach the tails and washed three times with schistosome culture medium 169 [25] supplemented by 100 U/ml penicillin (Lonza), 100 μg/ml streptomycin (Lonza) and 0.25 μg/ml amphotericin B (Lonza) in which they were cultivated at 37 °C and 5% CO_2_. After 2 days, these schistosomulum-like stages were washed three times with a sterile 0.1 M phosphate buffered saline (pH 7.2; PBS) and added to the cells.

Alternatively, LS were concentrated in 100 μl of PBS supplemented by cOmplete Mini protease inhibitors (Roche, Basel, Switzerland) and homogenized by a sonicator (3 × 30 s, amplitude 60 W, cooled on ice; Vibra Cell). To remove debris, the suspension was centrifuged 2 × 10 min at 16,000× *g* and the protein concentration in the supernatant (referred as soluble fraction of LS homogenate, HSF) was determined by Quant-iT Protein Assay Kit (Invitrogen). The samples were used immediately or stored at -80 °C.

Recombinant cathepsins B1.1 and B2 of *T. regenti* (rTrCB1.1 and rTrCB2) were expressed in the methylotrophic yeast *Pichia pastoris* (Invitrogen) as described previously [13, 18]. For purification of the recombinant cathepsins, Macro-Prep High S Support (Bio-Rad) and fast protein liquid chromatography (BioLogic HR system, Bio-Rad, Hercules, California, USA) were used. In order to dispose of excessive N-linked oligosaccharides, the purified recombinant enzymes were deglycosylated by Native protein deglycosylation kit (Sigma-Aldrich). Complete buffer exchange with sterile PBS was accomplished using Amicon Ultra 0.5 ml centrifugal filters (10.000 MWCO, Merck Millipore, Billerica, Massachusetts, USA). The concentration of proteins was then measured as described above. Prior to use in stimulation experiments, proteolytic activity of both cathepsins was tested using the fluorogenic peptide substrate Z-Phe-Arg-AMC (Bachem, Bubendorf, Switzerland) as described elsewhere [13, 18]. The samples were used immediately or stored at -80 °C.

Samples of HSF, rTrCB1.1 and rTrCB2 were tested for the presence of endotoxin contamination, which could skew the results of stimulation experiments, by *Limulus* amoebocyte lysate chromogenic endotoxin quantitation kit (detection limit: 0.1 EU/ml; Thermo Fisher Scientific) following the manufacturer’s instructions. In all samples, the endotoxin concentration was below 0.03 EU per microgram of the protein. When adjusted to the quantity of the particular stimulant added to the cells, the amount of endotoxin did not ever exceed 70 pg per well for HSF, 3 pg per well for rTrCB1.1, and 6 pg for rTrCB2 during the experiments.

### Measurement of NO production

NO production by astrocyte and microglia cultures was assessed by indirect measurement of nitrite concentration by colorimetric Griess assay in a 96-well plate (Nunc). Initially, 100 μl of the cell supernatant were mixed with the same volume of 58 mM sulphanilamide (Sigma-Aldrich) in 2.5% phosphoric acid and incubated for 10 min. Subsequently, 100 μl of 12 mM N-(1-naphthyl)ethylenediamine (Sigma-Aldrich) in 2.5% phosphoric acid were added and the samples were incubated for 10 min in the dark. The absorbance was then measured by a microplate reader (Tecan Infinite M200) at 550 nm. Standards of sodium nitrite in range of 100–3.125 μM (two-fold serial dilution) were used for calibration of the assay.

### Detection of inducible NO synthase (iNOS) by immunofluorescence

To examine iNOS expression in the cells surrounding dead parasites, immunofluorescence was employed. The samples were fixed by freshly prepared 4% paraformaldehyde for 15 min, treated by 5% NGS and 0.3% Triton X-100 in PBS for 60 min and left in rabbit monoclonal anti iNOS antibody (1:400 in 1% bovine serum albumin and 0.3% TritonX-100; Cell Signaling Technology, Danvers, Massachusetts) overnight at 4 °C. Finally, the samples were incubated with goat anti rabbit Alexa Fluor 594 (1:2000 in PBS; Thermo Fisher Scientific) and observed as described above. Murine RAW 264.7 macrophages stimulated by LPS (1 μg/ml) for 24 h were used as a positive control for iNOS expression.

### Measurement of cytokine production

Concentration of selected proinflammatory (IL-1β, IL-6 and TNF-α) and anti-inflammatory (IL-10, TGF-β1) cytokines was measured in supernatants of astrocyte and microglia cultures by sandwich enzyme-linked immunosorbent assay (ELISA) using ELISA MAX standard sets (BioLegend, San Diego, California, USA) for IL-1β, IL-6, and TNF-α or Platinum ELISA kits (eBioscience, San Diego, California, USA) for IL-10, TGF-β1 according to the manufacturer’s instructions. To assess the concentration of cytokines in samples, a calibration curve from recombinant cytokines was constructed. In blank samples, stock solution of cell culture medium was used and detection antibody and avidin-horseradish peroxidase were omitted; the absorbance of blank samples was subtracted from those of analyzed samples.

### Statistical analysis

All analyses were performed by GraphPad Prism, version 6. Data distribution of NO and cytokine concentrations and LS motility was tested for normality by Shapiro-Wilk test. Kruskall-Wallis (K-W) test followed by Dunn’s multiple comparisons test (NO and cytokines concentrations) or one-way analysis of variance (ANOVA) followed by Dunnett’s multiple comparisons test (LS motility) were used to determine which groups differed from the control ones. *P*-values lower than 0.05 were considered significant. Data are presented as mean values followed by the standard error of the mean (SEM).

## Results

### Cell culture purity

**Fig. 1:**
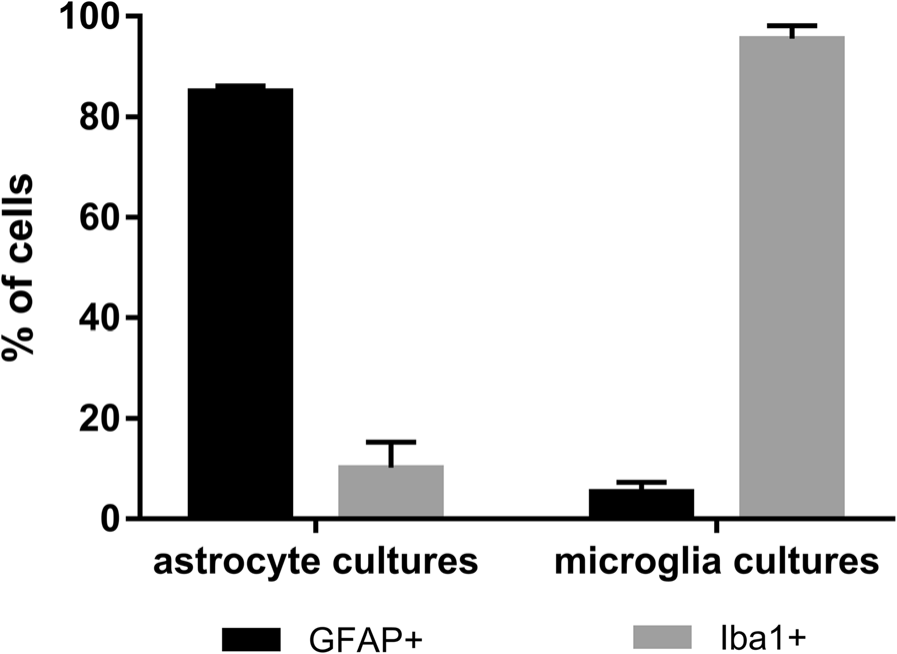
Purity of glial cell cultures used in the study as revealed by immunofluorescence. In astrocyte cultures, GFAP+ cells corresponding to astrocytes were dominant, while microglia cultures consisted prominently from Iba1+ cells corresponding to microglia. Data are shown as the mean ± standard error of the mean (SEM) of five independent samples

We applied indirect immunofluorescence to determine the purity of primary cell cultures used in the stimulation experiments (Fig. 1). In astrocyte cultures, 85.5 ± 0.4% of cells were GFAP+ and 10.2 ± 2.3% displayed Iba1+ signal. In microglia cultures, 95.5 ± 1.2% of cells were Iba1+ while 5.4 ± 0.8% exhibited GFAP+ signal. Overall, other cell types constituted less than 15% of total amount of cells in each culture and such contamination was formed predominantly by microglia in astrocyte cultures and vice versa.

### NO production

**Fig. 2:**
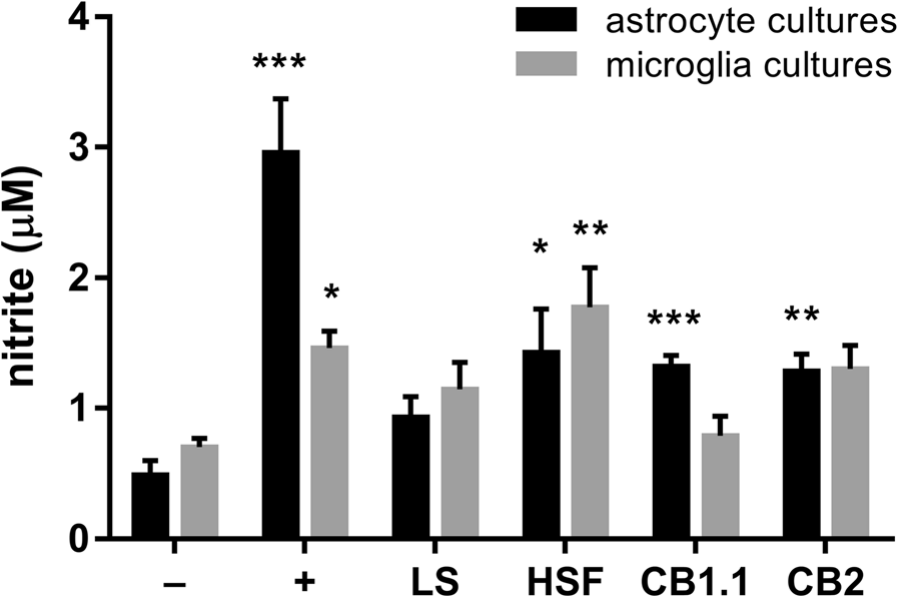
NO production by murine primary astrocyte and microglia cultures exposed to *Trichobilharzia regenti* derived stimulants. Griess assay was used to measure concentration of nitrite (NO degradation product) in cell supernatants 48 h after the cells were exposed to the stimulants. *Key*: −, non-stimulated group (a negative control); +, LPS - stimulated group (a positive control); LS, living schistosomulum-like stages stimulated group; HSF, soluble fraction of LS homogenate stimulated group; CB1.1, recombinant *T. regenti* cathepsin B1.1 stimulated group; CB2, recombinant *T. regenti* cathepsin B2 stimulated group. Data are shown as the mean ± standard error of the mean (SEM) of five independent experiments. Significant differences in comparisons with the non-stimulated group are marked by asterisks: *P < 0.05, **P < 0.01, ***P < 0.001 (Dunn’s multiple comparisons test)

Production of NO was monitored indirectly by measuring nitrite concentration in cell supernatants 48 h after the cell cultures were exposed to the stimulants; nitrites are products of NO oxidation. In case of astrocyte cultures, significant differences were noticed among the groups (K-W test: *χ* ^2^ = 36.00, *df* = 5, *P* < 0.001; Fig. 2, black bars). Particularly, in supernatants of astrocyte cultures exposed to HSF (*P* = 0.027), rTrCB1.1 (*P* < 0.001), and rTrCB2 (*P* = 0.001), about 2.6-fold elevated levels of nitrite were detected if compared to the non-stimulated group. Significant differences in nitrite concentrations were revealed also among microglia cultures (K-W test: *χ* ^2^ = 19.84, *df* = 5, *P* = 0.001; Fig. 2, grey bars), but the increase was recorded only in case of microglia cultivated with HSF (*P* = 0.004). When microglia were exposed to rTrCB2, the increase of nitrite concentration was not significant (*P* = 0.072).

**Fig. 3:**
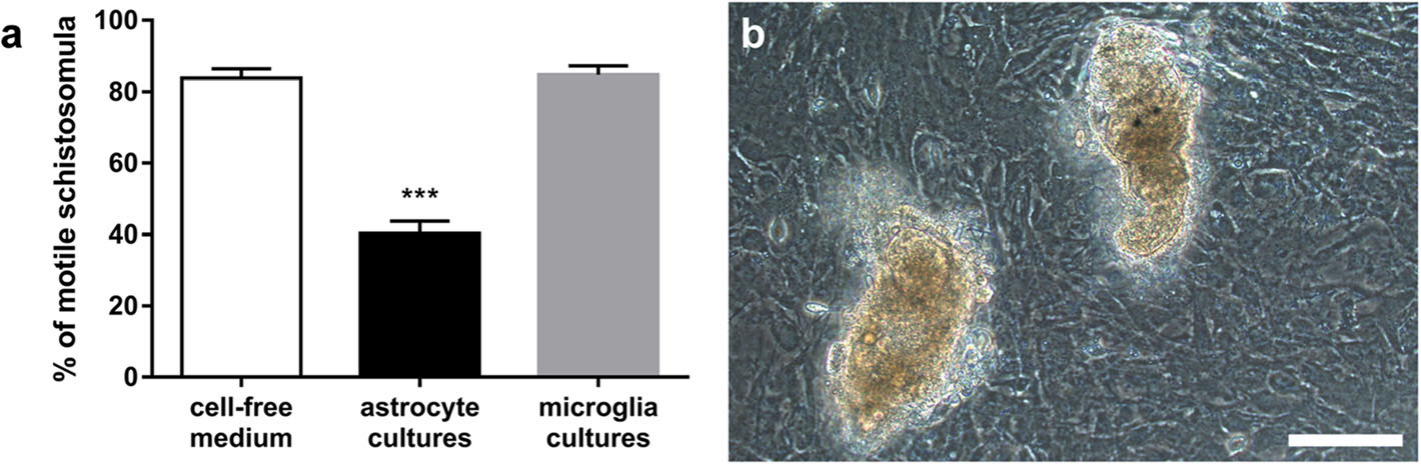
Motility of *Trichobilharzia regenti* schistosomulum-like stages grown 48 h with astrocyte and microglia cultures. **a** Significantly less parasites remained motile when cultivated in astrocyte cultures (***P < 0.001, Dunnett’s multiple comparisons test). Data are shown as the mean ± standard error of the mean (SEM) of five independent experiments. **b** Micrograph showing two schistosomula firmly attached to the cell layer in the astrocyte culture. *Scale-bar*: 100 μm

LS induced a rise of nitrite concentration in neither astrocyte (*P* = 0.093) nor microglia cultures (*P* = 0.419). However, we observed a significant effect of the type of the cell culture on parasite motility (ANOVA: *F*_(2,37)_ = 74.28, *P* < 0.001). In particular, markedly reduced motility of parasites dwelling in astrocyte cultures (*P* < 0.001), if compared to those cultivated in cell-free medium, was recorded (Fig. 3a). About a third of all parasites were even firmly attached to the cell layer (Fig. 3b). As revealed by immunofluorescence, no iNOS specific signal was detected in the cells surrounding the parasites. As a control for the correct immunofluorescence procedure, we succeeded to observe iNOS+ cells in LPS-stimulated RAW 264.7 macrophages (Additional file 2: Figure S2).

### Cytokine production

Production of IL-1β, IL-6, TNF-α, IL-10 and TGF-β1 was measured in supernatants of astrocyte and microglia cultures following 48 h of exposure to *T. regenti* derived stimulants.

**Fig. 4:**
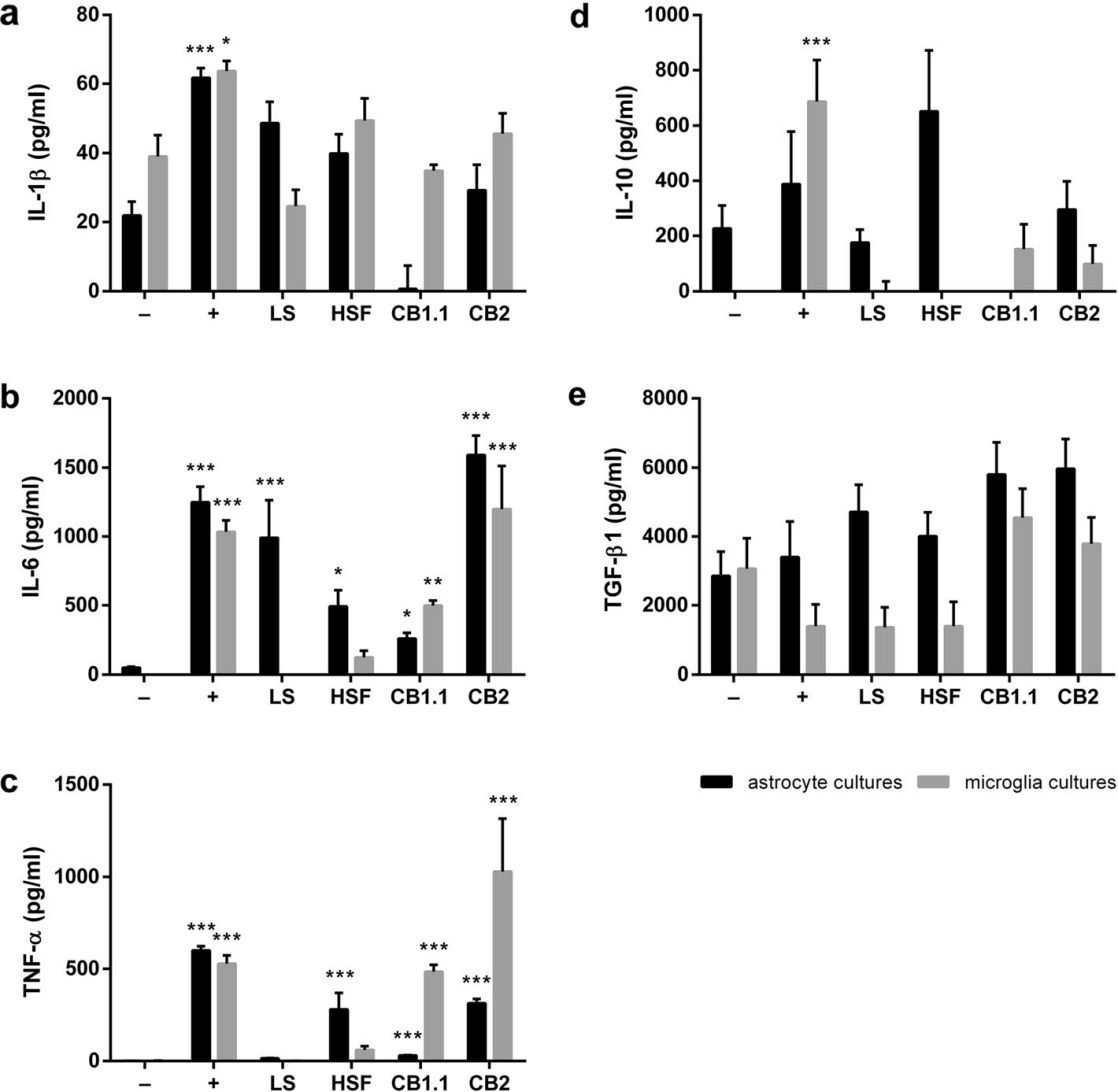
Cytokine production by murine primary astrocyte and microglia cultures exposed to *Trichobilharzia regenti* derived stimulants. Sandwich ELISA was used to measure the concentration of IL-1β (**a**), IL-6 (**b**), TNF-α (**c**), IL-10 (**d**), and TGF- β1 (**e**) in cell supernatants 48 h after the cells were exposed to the stimulants. *Key*: −, non-stimulated group (a negative control); +, LPS-stimulated group (a positive control); LS, living schistosomulum-like stages stimulated group; HSF, soluble fraction of LS homogenate stimulated group; CB1.1, recombinant *T. regenti* cathepsin B1.1 stimulated group; CB2, recombinant *T. regenti* cathepsin B2 stimulated group. Data are shown as the mean ± standard error of the mean (SEM) of five independent experiments. Significant differences in comparisons with the non-stimulated group are marked by asterisks: *P < 0.05, ** *P* < 0.01, ***P < 0.001 (Dunn’s multiple comparison test)

Although differences in IL-1β concentration were identified among both astrocyte (K-W test: *χ* ^2^ = 41.19, *df* = 5, *P* < 0.001; Fig. 4a, black bars) and microglia (K-W test: *χ* ^2^ = 21.62, *df* = 5, *P* < 0.001; Fig. 4a, grey bars) cultures undergoing different treatments, none of the *T. regenti* derived stimulants significantly increased IL-1β secretion in any of the cultures. Solely in case of astrocyte cultures exposed to HSF, the increment in IL-1β nearly reached a significant value (*P* = 0.055).

As for IL-6 concentration, significant differences were noticed among both astrocyte (K-W test: *χ* ^2^ = 56.33, *df* = 5, *P* < 0.001; Fig. 4b, black bars) and microglia (K-W test: *χ* ^2^ = 46.98, *df* = 5, *P* < 0.001; Fig. 4b, grey bars) cultures exposed to various stimulants. For astrocyte cultures, rTrCB2 appeared as the most potent inducer of IL-6 secretion (*P* < 0.001) being followed by LS (*P* < 0.001). HSF (*P* = 0.013) and rTrCB1.1 (*P* = 0.027) increased IL-6 production in astrocyte cultures as well, but to a lesser extent. Both rTrCB1.1 (*P* = 0.001) and rTrCB2 (*P* < 0.001) increased IL-6 levels also in microglia cultures.

Similarly to IL-6, highly significant differences in the concentration of TNF-α were observed among both astrocytes (K-W test: *χ* ^2^ = 66.28, *df* = 5, *P* < 0.001; Fig. 4c, black bars) and microglia (K-W test: *χ* ^2^ = 46.78, *df* = 5, *P* < 0.001; Fig. 4c, grey bars) cultures to which different treatments were applied. As for astrocyte cultures, HSF and rTrCB2 provoked similarly elevated TNF-α secretion (both *P* < 0.001). Likewise, after rTrCB1.1 treatment, significant (*P* < 0.001), but 10-fold lower increase in TNF-α concentration was detected. Both rTrCB1.1 and rTrCB2 also stimulated microglia cultures to produce TNF-α (both *P* < 0.001), the latter being a stronger trigger.

Differences in IL-10 production were observed among both astrocyte (K-W test: *χ* ^2^ = 21.25, *df* = 5, *P* < 0.001; Fig. 4d, black bars) and microglia (K-W test: *χ* ^2^ = 32.76, *df* = 5, *P* < 0.001; Fig. 4d, grey bars) cultures following different treatment. Nevertheless, no significant alterations of IL-10 concentration were recorded if the groups exposed to *T. regenti* derived stimulants were compared to the untreated control. In case of astrocyte cultures affected by rTrCB1.1, the decrease of IL-10 was not significant (*P* = 0.084). The same applies to apparent increment of IL-10 in microglia culture treated with rTrCB1.1 (*P* = 0.091) and rTrCB2 (*P* = 0.055).

Considering levels of TGF-β1, differences were found among astrocyte (K-W test: *χ* ^2^ = 11.61, *df* = 5, *P* = 0.040; Fig. 4e, black bars) and microglia (K-W test: *χ* ^2^ = 15.29, *df* = 5, *P* = 0.009; Fig. 4e, grey bars) cultures undergoing diverse treatment. However, significant changes of TGF-β1 concentration, if compared to the untreated group, were not noticed in any of the treated groups, including astrocyte cultures stimulated by rTrCB1.1 (*P* = 0.082) and rTrCB2 (*P* = 0.052).

## Discussion

In this study, we monitored the response of murine astrocytes and microglia after in vitro exposure to several *Trichobilharzia regenti* derived stimulants. Apart from its importance as the causative agent of cercarial dermatitis in humans [11], *T. regenti* is of a high interest just due to neuropathological manifestation of bird and mammalian infections [16]. However, the immune response in the CNS of infected mice has so far been described only by histopathological or immunohistochemical methods [17, 20, 21]. Since tissue inflammation correlating with presence of the parasite and axonal damage was observed in infected mice as early as seven dpi [17], we primarily focused on detection of proinflammatory cytokines and nitric oxide. These factors are known to be produced by activated astrocytes and microglia and may contribute to pathological processes [26–29].

*Trichobilharzia regenti* schistosomula appear in the murine CNS as soon as two dpi and persist there up to 3–4 weeks being localized predominantly in the white matter of the spinal cord [30]. Such ex vivo isolated living schistosomula and soluble fraction of their homogenate would definitely be the most natural stimulants of glial cells in vitro, simulating both initial (living parasites) and terminal (damaged parasites) phases of the infection. However, only around 5–15 schistosomula can be usually found in the CNS of an infected mouse [14, 20] with even decreasing yields during the long-term parasite passage in the laboratory [31]. Consequently, in order not to sacrifice excessive numbers of mice, LS for our stimulation experiments were prepared in vitro by mechanical transformation of cercariae. By this method, hundreds of schistosomulum-like stages resembling ex vivo schistosomula in terms of glycocalyx shedding, penetration glands emptying and immunoreactivity can be obtained [24, 32].

Living schistosomula are directly exposed to the host immune system in the CNS. Both tegumental antigens [32] and presumably parasite ESP are capable of triggering the host immune response. In this study, we demonstrated that in vitro LS induce only IL-6 secretion by exclusively astrocyte cultures. This suggests that astrocytes might be involved in triggering tissue inflammation in the early phase of *T. regenti* infection. Astrocytes are generally known to respond to the CNS injury by participation in the tissue repair [33]. This was shown, apart from *T. regenti* [17], also in several other neuroinfections caused by helminths [34–40]. However, they can be active players in maintaining host proinflammatory response due to IL-6 secretion as well, which was proved, e.g. in murine toxoplasmosis [41]. Additionally, astrocytes are likely involved in destruction of migrating schistosomula since we observed highly reduced parasite motility and even dead schistosomula adhered to the cell layer in astrocyte, but not microglia cultures after 48 h. Nevertheless, no significant increase of nitrite concentration was noticed in cell supernatants. Furthermore, we did not observe the presence of iNOS in the cells surrounding the worms. This excludes possibility of increased NO production in such microenvironment, i.e. just around dying/dead parasites which remain unrecognized in terms of total nitrite increment. Therefore, it appears that NO is not the major factor responsible for initial parasite destruction, although its deleterious effects on helminths have been demonstrated in vitro, e.g. for schistosomula of human schistosomes [42, 43]. Other compounds, such as reactive oxygen species, should be further examined in order to determine the factors responsible for parasite destruction. As for microglia, which were suggested to contribute significantly to parasite destruction [17], we did not observe increase in NO production by microglia cultures. However, their role in parasite destruction cannot be completely excluded, since in vivo they might undergo activation leading to NO production elicited by factors (e.g. IL-1β, IL-12 or interferon-γ) secreted by other immune cells being presumably present in the inflamed tissue [44].

Apart from the exposure to LS, responsiveness of astrocytes and microglia to HSF was tested; HSF contains a mixture of parasite antigens, both surface and intrinsic, released from the damaged schistosomula. Contrary to LS, HSF triggered a significant production of NO in both astrocyte and microglia cultures. Similar effect (demonstrated at the level of enhanced iNOS expression) was shown for murine microglia treated in vitro with larval soluble antigen of the neurotropic nematode *Angiostrongylus cantonensis* [45]. As for *T. regenti*, NO production by glial cells might be associated with the in vivo occurring axonal damage [46, 47]. It was proposed that the axonal damage detected in both immunocompetent and immunodeficient (SCID) mice infected by *T. regenti* is caused mechanically by migrating schistosomula, and not by the host immune response [17]. However, our in vitro experiments show that astrocytes and microglia are capable of NO release after exposure to HSF suggesting that they can be responsible for NO-mediated nervous tissue pathology occurring in later phases of the infection when the worms are damaged and both surface and intrinsic antigens (contained in HSF) diffuse into the nervous tissue. Furthermore, astrocyte cultures were shown to secrete IL-6 and TNF-α after exposure to HSF. We hypothesize that this might contribute to the ongoing nervous tissue inflammation which progressively develops during the infection [17]. Interestingly, microglia cultures did not significantly raise production of IL-6 and TNF-α following exposure to HSF, although they are generally considered proinflammatory cells in the CNS. For example, they were suggested to be crucial players in mediating inflammation in murine cerebral angiostrongylosis [48]. However, no IL-6 and TNF-α secretion was detected after murine microglia were grown in the medium containing soluble factors from *Mesocestoides corti* metacestodes which are used as a murine model for neurocysticercosis [49].

Additionally, stimulatory properties of *T. regenti* recombinant cathepsins B1.1 and B2 were also tested in our experiments. These proteases are highly expressed in migrating schistosomula [50, 51] and can get in contact with the adjacent nervous tissue either when the parasite regurgitates (TrCB1.1 as a digestive enzyme, [18]) or when it struggles through the tissue (TrCB2 as a neurohistolytic enzyme, [13]). In our recent work [17] it remained questionable if secretions of schistosomula may be involved in the CNS pathogenesis, particularly in activation of glial cells. Our in vitro observations confirmed the capability of rTrCB1.1 and rTrCB2, components of parasite ESP, to strongly enhance production of IL-6 and TNF-α by both astrocyte and microglia cultures, and NO secretion in astrocyte cultures. Just in case of astrocyte cultures stimulated by rTrCB1.1, it cannot be excluded that the only slight increase in TNF-α concentration might have been caused by residual microglial cells. Nevertheless, in any case, this suggests that *T. regenti* cathepsins B1.1 and B2 may be involved in activation of astrocytes and/or microglia leading subsequently to promotion of the nervous tissue inflammation and possibly to the axonal damage (as mentioned before). Similarly, cathepsin B of *A. cantonensis* was proposed to be involved in mouse CNS invasion and immunomodulation; however, no functional assay was performed to verify this hypothesis [52]. On the contrary, cathepsin-containing ESP of *Paragonimus westermani* did cause rat microglia to produce NO in vitro [53], and this action was affirmed in vivo when ESP microinjection into the rat brain elicited activation of microglia and iNOS expression in the injured site [35]. Pertinent observation of immune effects of parasite ESP was recently reported also for cathepsin-containing ESP of *Naegleria fowleri*. They stimulated murine BV-2 microglial cells to secrete IL-1α and TNF-α which were suggested to contribute to inflammatory response occurring in the CNS during *N. fowleri* infection [54].

Finally, we evaluated whether astrocytes and microglia also produce anti-inflammatory cytokines IL-10 and TGF-β1 which could moderate the course of inflammation and reduce excessive tissue pathology. Microglia were shown to secrete IL-10 and TGF-beta during toxoplasmosis which attenuated the inflammatory immune response and led to asymptomatic persistence of the infection [41, 55, 56]. Significantly more astrocytes positive for TGF-β1 were observed in brains of patients suffering from cerebral malaria [57]. IL-10 and/or TGF-β1 were also detected in brains of individuals with neurocysticercosis [58] or neurotoxocarosis [59], but their cellular source was not identified. In our study, we did not observe any significant changes in secretion of these cytokines in either astrocyte or microglia cultures. This suggests that *T. regenti* derived stimulants do not exhibit conspicuous immunomodulatory effects in terms of IL-10 and TGF-β1 secretion by astrocytes and microglia.

## Conclusions

The presented study demonstrated in vitro the capability of primary murine astrocytes and microglia to secrete nitric oxide and proinflammatory cytokines IL-6 and TNF-α after exposure to the neuropathogenic schistosome *Trichobilharzia regenti*. The study evaluates immunogenic properties of particular parasite derived stimulants by performing functional assays using the relevant immunocompetent cells. Altogether, our data show that both astrocytes and microglia are able to recognize various stimulants of parasite origin and trigger secretion of the aforementioned immune factors. This suggests astrocytes and microglia might be active players responsible for the nervous tissue inflammation and pathogenesis during the infection of mice by *T. regenti*.

## Additional files

Additional file 1: Figure S1. Glial cell yields from neonatal murine brains. Graphical representation of the average number of obtained astrocytes and microglia adjusted to one brain. (DOCX 48 kb)
Additional file 2: Figure S2. Detection of iNOS in LPS-stimulated RAW 264.7 macrophages. Results from a positive control immunofluorescence experiment. (DOCX 971 kb)

## Abbreviations

ANOVA: Analysis of variance
CB1.1: Recombinant *Trichobilharzia regenti* cathepsin B1.1 (shortened version in graphs)
CB2: Recombinant *Trichobilharzia regenti* cathepsin B2 (shortened version in graphs)
CNS: Central nervous system
dpi: day(s) post-infection
ELISA: Enzyme-linked immunosorbent assay
ESP: Excretory/secretory products
GFAP: Glial fibrillary acidic protein
HSF: Soluble fraction of living schistosomulum-like stages of *Trichobilharzia regenti* homogenate
Iba1: Ionized calcium-binding adapter molecule 1
IL-1β: Interleukin 1β
IL-6: Interleukin 6
IL-10: Interleukin 10
iNOS: Inducible nitric oxide synthase
K-W test: Kruskall-Wallis test
LPS: Lipopolysaccharide
LS: Living schistosomulum-like stages of *Trichobilharzia regenti*
NGS: Normal goat serum
NO: Nitric oxide
PBS: Phosphate buffered saline
rTrCB1.1: recombinant *Trichobilharzia regenti* cathepsin B1.1
rTrCB2: recombinant *Trichobilharzia regenti* cathepsin B2
SEM: Standard error of the mean
TGF-β1: Transforming growth factor β1
TNF-α: Tumor necrosis factor
