# Supplementary material for: Nitric oxide and cytokine production by glial cells exposed in vitro to neuropathogenic schistosome Trichobilharzia regenti
Source: Parasit Vectors. 2016 Nov 14;9:579. doi: 10.1186/s13071-016-1869-7 (PMC5109812; doi:10.1186/s13071-016-1869-7)

**Additional file 1: Figure S1.** Glial cell yields from neonatal murine brains. From mixed glial cell cultures, primary astrocytes and microglia cultures were obtained. If counted and adjusted to one murine brain used for cell culture establishment, the yield of live astrocytes was 7.7-fold higher than that of live microglia as shown in the figure bellow. The cells were counted by Countess™ Automated Cell Counter (Invitrogen). Data are presented as mean values from 5 independent experiments followed by the standard error of the mean.


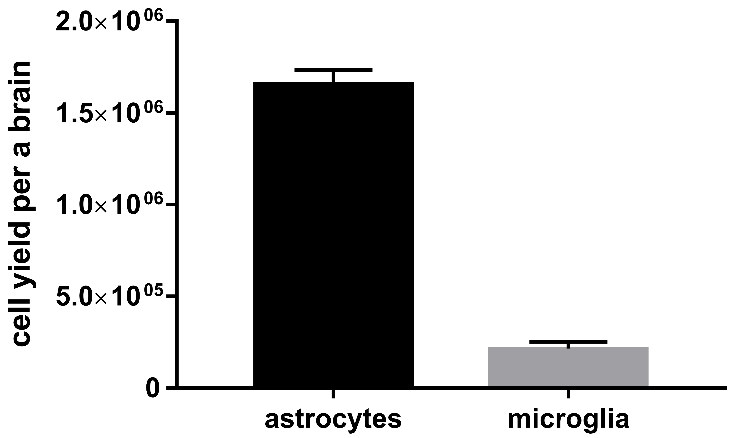

Supplement: Additional file 1: Figure S1. — Glial cell yields from neonatal murine brains. Graphical representation of the average number of obtained astrocytes and microglia adjusted to one brain. (DOCX 48 kb) [file 13071_2016_1869_MOESM1_ESM.docx]
