# Supplementary material for: Nitric oxide and cytokine production by glial cells exposed in vitro to neuropathogenic schistosome Trichobilharzia regenti
Source: Parasit Vectors. 2016 Nov 14;9:579. doi: 10.1186/s13071-016-1869-7 (PMC5109812; doi:10.1186/s13071-016-1869-7)

**Additional file 2: Figure S2.** Detection of iNOS in LPS-stimulated RAW 264.7 macrophages. Results from a positive control immunofluorescence experiment to confirm correctness of the procedure. iNOS signal (red) was detected in LPS-treated cells (a). No such signal was detected if the anti iNOS primary antibody was omitted in the protocol (b) or if the cells were not treated by LPS (c). The blue signal stands for nuclei stained by DAPI. Scale-bar: 20 μm.


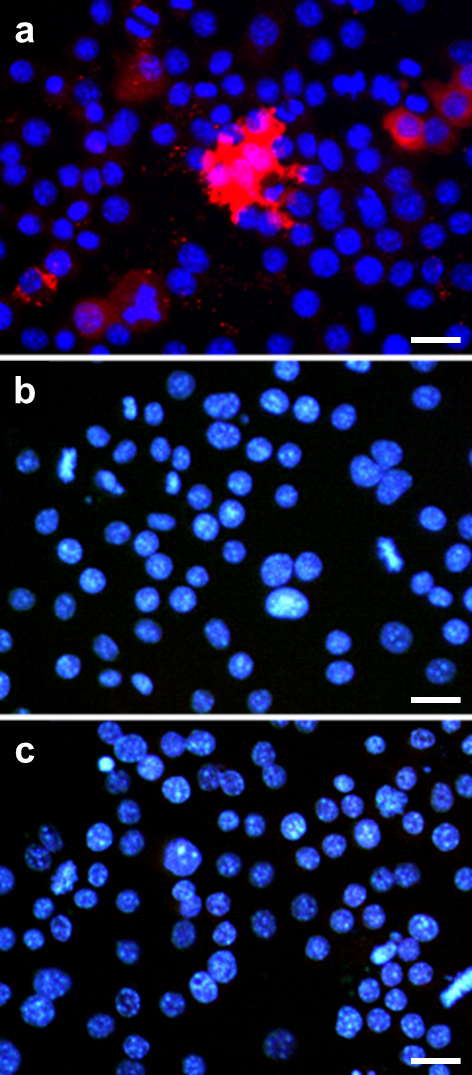

Supplement: Additional file 2: Figure S2. — Detection of iNOS in LPS-stimulated RAW 264.7 macrophages. Results from a positive control immunofluorescence experiment. (DOCX 971 kb) [file 13071_2016_1869_MOESM2_ESM.docx]
